# Involvement of Akt/NF-κB pathway in antitumor effects of parthenolide on glioblastoma cells in vitro and in vivo

**DOI:** 10.1186/1471-2407-12-453

**Published:** 2012-10-05

**Authors:** Hiromichi Nakabayashi, Keiji Shimizu

**Affiliations:** 1 Human Biology, Department of Health Sciences, Oita University of Nursing and Sciences, 2944-9 Megusuno, Oita, 870-1201, Japan; 2 Department of Neurosurgery, Kochi Medical School Kochi University, 783-8505, Nankoku, Japan

**Keywords:** Parthenolide, Glioblastoma, NF-κB, Invasion, Angiogenesis

## Abstract

**Background:**

Glioblastoma is the most common and most aggressive form of malignant glioma and is very difficult to treat. Controlling tumour cell invasion and angiogenesis is essential to improve the prognosis of glioblastoma patients. Since constitutive activation of nuclear factor-κB (NF-κB) is necessary for tumour progression, NF-κB may be an important pharmacological target for this disease. Our study aimed to evaluate the antitumour effects of parthenolide, a NF-κB inhibitor, in two human glioblastoma cell lines (U87MG and U373) and in glioblastoma xenografts. Furthermore, we aimed to investigate the molecular mechanisms underlying these effects.

**Methods:**

The anti-invasive and anti-angiogenic effects of parthenolide were analysed using in vitro invasion and angiogenesis assays. Parthenolide-induced growth inhibition of glioblastoma cells in vitro was determined using the MTT (methyl thiazolyl tetrazolium) assay. In addition, the effect of parthenolide on orthotropic implantation in vivo was evaluated using an intracerebral human glioblastoma xenograft model.

**Results:**

We found that parthenolide suppresses proliferation, invasion, and tumour- induced angiogenesis of glioblastoma cells. Molecular studies demonstrated that parthenolide suppresses gene and protein expression of angiogenic factors. Furthermore, parthenolide reduced Akt phosphorylation and activated mitochondrial signalling, suggesting that the antitumour function of parthenolide may be mediated not only by the inhibition of NF-κB but also by the inhibition of Akt signalling and the activation of apoptotic proteins. Parthenolide suppressed neovascularity and tumour growth in glioblastoma xenografts.

**Conclusion:**

The present study identified parthenolide as a new therapeutic agent for glioblastomas.

## Background

The serine/threonine kinase Akt, a downstream effector of phosphatidylinositol 3-kinase (PI3K), is involved in cell survival and anti-apoptotic signalling [1]. In a variety of human tumours, including glioblastoma, Akt is constitutively activated due to mutation or deletion of the phosphatase and tensin homologue (PTEN), a major negative regulator of the PI3K/Akt signal pathway [2,3]. The PTEN mutation frequency in glioblastoma is reported to be as high as 80% [3]. Recent studies have demonstrated that PI3K/Akt induction of cell survival signals is mediated, in part, through the activation of the nuclear factor kappa B (NF-κB) transcription factor [4]. NF-κB is linked to various signal transduction pathways and to transcription activation events that mediate cell proliferation, cell migration, and angiogenesis [5,6]. Among NF-κB-regulated genes [7], matrix metalloproteinase (MMP)-9 is closely associated with tumour invasion and tumour-induced angiogenesis, and vascular endothelial growth factor (VEGF) promotes tumour-induced angiogenesis. Therefore, inhibition of NF-κB may serve as an effective treatment strategy for many tumours through MMP-9 and VEGF downregulation and disruption of Akt signalling.

Parthenolide, a major sesquiterpene lactone from the plant *Tanacetum parthenium*, inhibits NF-κB both indirectly, by inhibiting the IκB kinase (IκK), and directly, by modifying p65 at a key cysteine residue in its activation loop [8,9]. Due to its anti-inflammatory and low toxicity properties, parthenolide has been used to treat migraine and rheumatoid arthritis [10]. In addition, many studies have investigated the effect of parthenolide treatment on human malignancies [5-7,9], although only two have examined the effect of parthenolide on glioblastoma cell proliferation in vitro [11,12]. However, their results were contradictory. Anderson et al. reported that parthenolide inhibition of glioblastoma cell proliferation is independent of the NF-κB pathway [11]. In contrast, Zanotto-Filho et al. reported that parthenolide-induced glioblastoma cell death is mediated by NF-κB inhibition [12]. We, therefore, aimed to clarify the mechanism involved in parthenolide inhibition of glioblastoma cell proliferation in vitro, and to examine the in vivo effect of parthenolide on tumour growth by using a xenograft model of glioblastoma. We also aimed to evaluate the effect of parthenolide on invasion capacity and tumour-induced angiogenesis in the PTEN-mutant human glioblastoma cell lines U87MG [13] and U373 [14]. To the best of our knowledge, this study is the first to investigate the antitumour effects of parthenolide on human glioblastoma in vivo, and to define the anti-invasive and anti-angiogenic effects of parthenolide treatment on glioblastoma cells in vitro.

## Methods

### Cell cultures and drug

PTEN-mutant human glioblastoma cell lines, U87MG and U373, were obtained from the American Type Culture Collection (Rockville, MD). Glioblastoma cells were maintained in Dulbecco’s modified Eagle’s medium (DMEM; Gibco, Gaithersburg, MD) supplemented with 10% foetal bovine serum (FBS; Gibco), penicillin (100 unit/mL), and streptomycin (100 mg/mL). Human brain microvascular endothelial cells (HBMECs) were purchased from Science Cell Research Laboratories (Carlsbad, CA) and maintained in RPMI 1640 (Gibco) supplemented with 10% FBS, 10% NuSerum (BD Bioscience, Mountain View, CA), modified Eagle’s medium nonessential amino acids (1%) and vitamins (1%) (Gibco), sodium pyruvate (1 mM), and EC growth supplement (30 μg/mL). Culture flasks were coated with 0.2% type-I collagen to support the growth of HBMEC monolayers, and experiments were performed using passages 3–10. All cells were cultured at 37°C in a humidified atmosphere containing 5% CO_2_.

Parthenolide was purchased from Sigma-Aldrich (St. Louis, MO), dissolved in dimethyl sulphoxide (DMSO) to a concentration of 10 mmol/L and stored in the dark at −80°C. A sample of DMSO was stored under the same conditions and used as a control treatment.

### ELISA based NF-κB transcription factor activity assay

Glioblastoma cells were treated with different concentrations (0.1–50 μM) of parthenolide for 48 h, and then nuclear extracts were analysed using the TransAM NF-κB p65 transcription factor assay kit (Active Motif, Carlsbad, CA), according to the manufacturer’s recommendations. NF-κB complexes were captured by binding to a consensus 5′-GGGACTTTCC-3′ oligonucleotide immobilised on a 96-well plate. Bound NF-κB was quantified by incubating with anti-p65 primary antibody followed by horseradish peroxidase (HRP)-conjugated goat anti-rabbit IgG and spectrophotometric detection at a wavelength of 450 nm. Data were expressed as the percentage of NF-κB/DNA binding relative to control cells. The assay was conducted three times for each cell line.

### Methyl thiazolyl tetrazolium (MTT) assay

Glioblastoma cells (1 × 10^4^ cells) were plated into 96-well plates in 100 μL of DMEM containing 10% FBS. HBMECs (1 × 10^4^ cells) were plated into 96-well plates in 100 μL of EGM-2 medium (Kurabo, Osaka, Japan) containing 2% FBS. After 24 h, parthenolide was added to each well at the indicated concentrations (0.1–50 μM). After 24 h or 48 h, 50 μL of MTT (2 mg/mL) was added to each well and incubated at 37°C for a further 3 h. The 570 nm absorbance of the dissolved precipitate was measured using a microplate reader. The cell proliferation index was calculated as the ratio of the absorbance of parthenolide-treated cells to that of control cells. The assay was conducted three times for each cell line.

### *In vitro* invasion assay

In vitro invasion assay was performed using Transwell invasion chambers (BioCoat; BD Biosciences). Glioblastoma cells were cultured in 24-well plates. An insert was used to divide each well of the plate into lower and upper chambers. The bottom of the insert comprised an 8.0-μm pore size PET membrane coated with Matrigel (BD Biosciences). The lower chamber was filled with 700 μL of DMEM supplemented with 0.1% bovine serum albumin (BSA) culture medium and human fibronectin (12.5 μg/mL). Subconfluent glioblastoma cells were harvested and resuspended in 500 μL of DMEM supplemented with 0.1% BSA containing parthenolide (1–50 μM), and 5.0 × 10^4^ cells/well were added to the upper chamber. After incubation for 23 h, the cells on the upper surface of the filters were removed with cotton swabs. Cells on the lower surface of the filters were fixed using 70% ethanol, stained with Giemsa stain, and five randomised fields were counted at 200× magnification. The assay was conducted three times for each cell line.

### Tube formation assay using a HBMEC-glioblastoma cell co-culture method

A modified tube formation assay using culture inserts was used to assess in vitro angiogenesis. Culture plates (24-well) were coated with 300 μL/well Matrigel (BD Biosciences) and polymerised at 37°C for 30 min. HBMECs in 5% EBM-2 medium containing FBS were plated (8 × 10^4^ cells/well) into the coated wells, and an insert plate containing a 1-μm pore PET membrane (Falcon HTS Multiwell Insert Systems, BD Biosciences) was placed onto the plate. U87MG cells or U377 cells were also cultured in the insert plate to allow soluble angiogenic factors secreted from the glioblastoma cells to reach the endothelial cells in the lower chambers. Parthenolide (2.5-50 μM) was added to the culture medium. After 7 days, four randomly selected fields of cells from each treatment were digitally photographed, and the length of the capillary-like structures within the gel matrix was measured using ImageJ software (http:// rsb.info.nih.gov/ij/). The assay was conducted three times for each cell line.

### Western blot analysis

Akt phosphorylation was analysed in glioblastoma cells by western blotting. Glioblastoma cells were starved in serum-free condition for 24 h, stimulated with 5 ng/mL PMA for 45 min, and then treated with parthenolide (2.5–10 μM) for 2 h. Control cells were not stimulated with PMA. Cells were harvested in buffer containing 50 mM Tris–HCl (pH 7.4), 150 mM NaCl, 1 mM EDTA, 1% (v/v) Triton X-100, and protease and phosphatase inhibitors (Sigma– Aldrich). Protein concentration was measured using the Bradford assay with a BSA standard. Cell lysate samples (50 μg) were separated by 10% SDS-PAGE and transferred onto nitrocellulose membranes. Membranes were incubated overnight at 4°C with anti-phospho-Akt (Ser473) rabbit antibody (Cell Signalling Technology, Danvers, MA) or total Akt rabbit antibody (Cell Signalling Technology), incubated with HRP-conjugated anti-rabbit IgG sheep antibody (GE Healthcare, Piscataway, NJ) for 1 h at room temperature, and then, the proteins were visualised using a ECL^+^ Chemiluminescence kit (GE Healthcare).

To analyse the effect of parthenolide on Bcl-2 family protein expression and caspase activation, glioblastoma cells were in serum-free condition starved for 24 h, and then treated with parthenolide (5 μM) for 6 h, 12 h, or 24 h. Bcl-2 family protein expression was assessed by western blotting using anti-Bcl-2 rabbit antibody (Cell Signalling Technology), anti-Bak rabbit antibody (Cell Signalling Technology), and anti-Bax rabbit antibody (Cell Signalling Technology) primary antibodies. Caspase activation was assessed using rabbit anti-caspase 3 (Cell Signalling Technology), rabbit anti-cleaved caspase 3 (Cell Signalling Technology), rabbit anti-caspase 9 antibody (Cell Signalling Technology), and rabbit anti-cleaved caspase 9 (Cell Signalling Technology) antibodies.

β-Actin expression, detected using mouse anti-β-actin monoclonal antibody (Sigma-Aldrich), was used as a loading control. Western blot experiments were performed at least twice, and protein expression was determined by quantitative densitometry.

### Evaluation of VEGF and MMP-9 gene expression

The effect of parthenolide on VEGF and MMP-9 gene expression was analysed by semi-quantitative RT-PCR. Total cellular RNA was extracted from parthenolide-treated (2.5, 5, or 10 μM; 12 h) glioblastoma cells using an RNeasy Kit (Qiagen, Valencia, CA) according to the manufacturer’s instructions. RT-PCRs were performed using a Takara RNA PCR kit (AMV) ver. 3.0 (Takara, Shiga, Japan) according to the manufacturer’s protocol. PCR amplification was performed with the following primers: VEGF forward, 5′-CCATGA ACTTTCTGCTGTCTT-3′; VEGF reverse, 5′-TCGATCGTTCTGTATCAGTCT- 3; MMP-9 forward, 5′-GAATTCAGAACCAATCTCGACAGGCA-3′; MMP-9 reverse, 5′-GAATCC AGA ACCAATCTCACCGACAGGCA-3′. As an internal control, a human GAPDH fragment was amplified using the following primers: forward, 5′-TGTTGCCATCAATGACCC-3′; and reverse, 5′-GCAGAGATGATGACCCTT-3′. RT-PCR was performed in at least two independent experiments.

### VEGF and MMP-9 secretion from glioblastoma cells

To determine the effect of parthenolide on the secretion of VEGF and MMP-9 from glioblastoma cells, VEGF and MMP-9 protein levels were measured in the culture medium of parthenolide-treated (5 or 10 μM; 12 h) glioblastoma cells by using a VEGF and MMP-9 ELISA kit (GE Healthcare). Data from parthenolide-treated cells were expressed as percentage of untreated controls, and the ELISA assay was conducted three times for each cell line.

### Effect of parthenolide on orthotopic cell transplantation

Athymic female mice (BALB/c *nu/nu*), aged 6–8 weeks, were obtained from Charles River Japan (Atsugi, Japan). Mice were anaesthetised with an intraperitoneal injection of sodium pentobarbital (60 mg/kg), and then injected intracerebrally with U87MG cells (1 × 10^5^) through a small hole drilled 2 mm anterior and 2 mm lateral to the bregma. Starting immediately after tumour cell transplantation, parthenolide (10 mg·kg^-1^·day^-1^) was administered intraperitoneally to mice (n = 10) every day. Control mice (n = 10) were treated with DMSO vehicle. All mice were sacrificed on day 22, and their brains were excised and snap-frozen in liquid nitrogen. Growth of intracerebral tumours was confirmed by histological evaluation. Serial coronal sections (30 μm) were cut using a cryo-microtome from the rostral to the caudal edge of tumour-containing brain tissue. Tumour size was estimated in sequential tumour sections by using an Image-Pro system (Media Cybernetics Inc., Silver Spring, MD). All procedures involving animals were approved by the animal care committee of Kochi University and were done in accordance with institutional and Japanese government guidelines for animal experiments.

### Histological assessment of xenograft tumours

For immunohistological examination of xenograft tumours, mice were treated as described above, and their brains were harvested, fixed in buffered formalin, and embedded in paraffin. Paraffin sections (4 μm) were deparaffinised and dehydrated. MVD was assessed by counting the number of microvessels in one microscope field (at 200× magnification) following haematoxylin-eosin (HE) staining. Immunohistochemical staining for VEGF and MMP-9 was done by incubating paraffin sections with anti-VEGF (Abcam Inc., Cambridge, MA) and anti-MMP-9 (Abcam Inc.) antibodies, followed by incubation with HRP (DAKO, Glostrup, Denmark) by using the EnVision^+^ System, and detection with diaminobenzidine. The percentage of VEGF and MMP-9 immunoreactive cells was determined in 10 high-power microscopy fields.

### Statistical analysis

The statistical significance of ELISA data was analysed using an unpaired Student’s t-test. The results of invasion and angiogenesis assays were statistically analysed by one-way analysis of variance (ANOVA). The statistical significance of differences in tumour volume between control and treated mice in the orthotopic implantation model was analysed using unpaired and paired Student’s t-tests. For all statistical analyses, a p value of <0.05 was considered to be statistically significant. All values are presented as mean ± standard deviation (SD).

## Results

### Parthenolide suppresses NF-κB transcriptional activity and inhibits tumour cell proliferation

**Figure 1:**
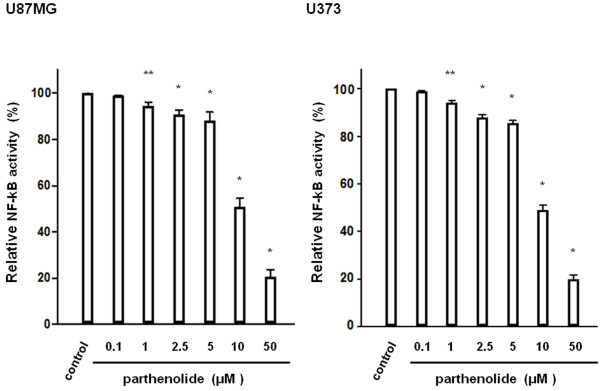
**NF-κB transcription factor activity.** NF-κB transcriptional activity was analysed in glioblastoma cells 48 h after treatment with parthenolide by using an ELISA-based assay. The assay showed that the transcriptional activity of NF-κB in the two glioblastoma cell lines decreased as the concentration of parthenolide increased. Data is presented as mean ± SD (n = 3). Points indicate bars + SD; * indicates p < 0.0001; ** indicates p < 0.01.

**Figure 2:**
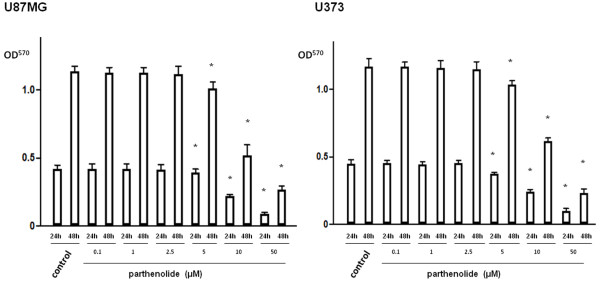
**MTT assay.** Glioblastoma cells were treated with parthenolide, and cell proliferation was assessed using the MTT assay. Parthenolide inhibited the growth of glioblastoma cells at concentrations above 5 μM in a dose-dependent manner. Data is presented as mean ± SD (n = 3). Points indicate bars + SD; * indicates p < 0.0001.

In two glioblastoma cell lines treated with parthenolide, we found that NF-κB transcriptional activity decreased as parthenolide concentration increased (Figure 1; *, p < 0.0001; **, p < 0.01). Further, we used an MTT assay to investigate the effect of parthenolide treatment on glioblastoma cell proliferation, and found that parthenolide concentrations above 5 μM suppress the growth of glioblastoma cells in a dose-dependent manner (Figure 2; *, p < 0.0001).

### Parthenolide suppresses tumour cell invasion and tumour-induced angiogenesis in vitro

**Figure 3:**
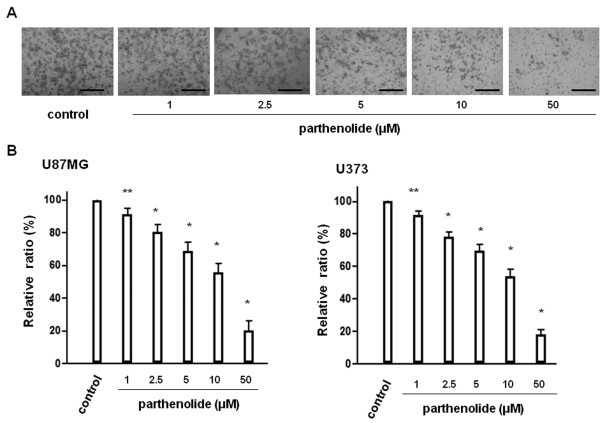
**Matrigel invasion assay.** Glioblastoma cells were treated with parthenolide, and cell invasion was assessed using a Matrigel invasion assay. (**A**) Representative U87MG cell data. (**B**) Number of invading glioblastoma cells relative to control. The number of invaded cells decreased as the concentration of parthenolide increased. Bar = 100 μm. Data is presented as mean ± SD (n = 3). Points indicate bars + SD; * indicates p < 0.0001; ** indicates p < 0.01.

**Figure 4:**
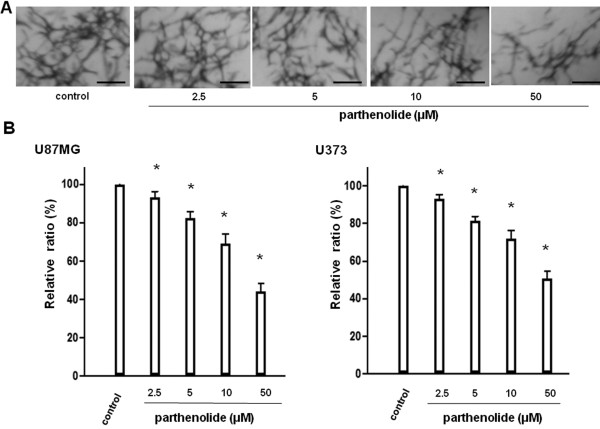
***In vitro *angiogenesis assay.** Glioblastoma cells were treated with parthenolide, and in vitro angiogenesis was assessed using a modified tube formation assay. (**A**) Representative tube formation data. (**B**) Tube formation was significantly suppressed by parthenolide in a dose-dependent manner. Data is presented as mean ± SD (n = 3). Points indicate bars + SD; * indicates p < 0.0001. Bar = 5 mm.

We evaluated the inhibitory effect of parthenolide on tumour cell invasion by using U87MG cells (Figure 3A), and observed that the numbers of invading cells decreased as parthenolide concentration increased. Therefore, glioblastoma cell invasion capacity is significantly suppressed by parthenolide (Figure 3B; *, p < 0.0001; **, p < 0.01). We next evaluated the effect of parthenolide on tumour-induced angiogenesis by using the same cell line (Figure 4A), and found that increasing parthenolide concentrations resulted in a significant decrease in tube length relative to control cells (Figure 4B; *, p < 0.0001).

### Parthenolide inhibits Akt phosphorylation and Bcl-2 expression and induces caspase activation and Bak and Bax expression

**Figure 5:**
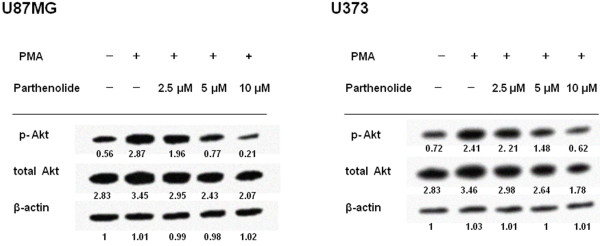
**Akt Phosphorylation.** Glioblastoma cells were stimulated with PMA prior to parthenolide treatment, and Akt phosphorylation was analysed by western blotting. Representative blots are shown. Akt expression and phosphorylation in both glioblastoma cell lines was stimulated by PMA, but suppressed by parthenolide in a dose-dependent manner.

**Figure 6:**
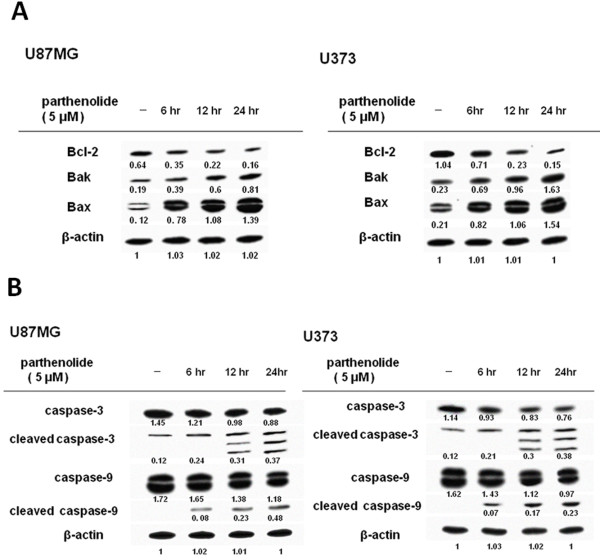
**Apoptosis-related protein expressions.** The expression of apoptotic proteins in glioblastoma cells stimulated with PMA prior to parthenolide treatment was assessed by western blotting. Representative blots are shown. (**A**) Analysis showed that parthenolide decreased the protein expression of Bcl-2 and increased the protein expression of Bak and Bax in a dose-dependent fashion. (**B**) Analysis showed that parthenolide induced the activation of caspase-3 and −9 in a dose-dependent manner.

We observed that a low basal level of Akt phosphorylation was present in glioblastoma cells in the absence of PMA treatment, but that Akt phosphorylation was strongly increased following PMA stimulation. However, parthenolide treatment inhibited PMA-induced Akt phosphorylation in a dose-dependent manner (Figure 5). Furthermore, treatment with parthenolide inhibited Bcl-2 protein expression, and increased Bak and Bax protein expression (Figure 6A) and caspase-3 and caspase-9 activation (Figure 6B) in a dose-dependent manner.

### Parthenolide suppresses VEGF and MMP-9 gene and protein expression

**Figure 7:**
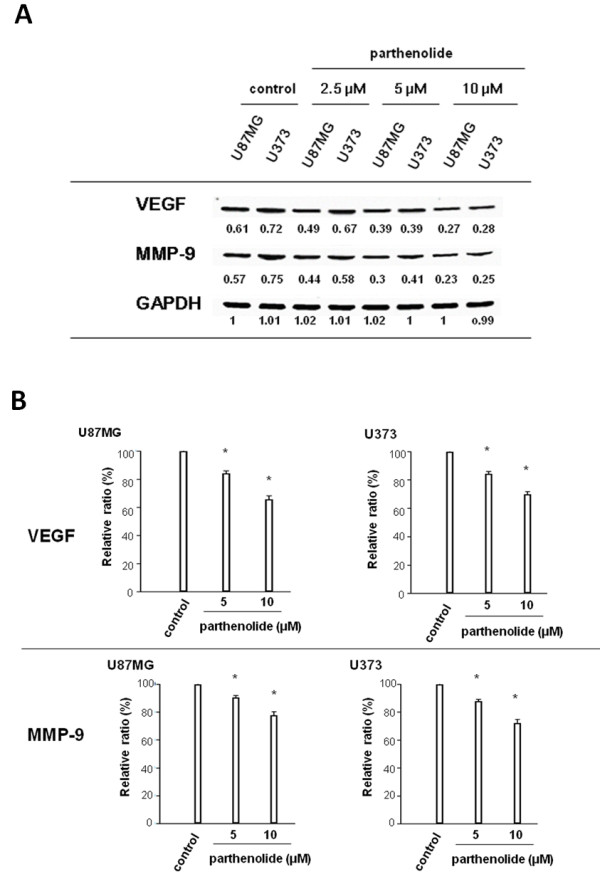
**Expression of MMP-9 and VEGF.** (**A**) Glioblastoma cells were treated with parthenolide, and MMP-9 and VEGF gene expression was analysed by semi-quantitative RT-PCR. Parthenolide attenuated the mRNA expression of MMP-9 and VEGF compared with the control. (**B**) Secretion of MMP-9 and VEGF protein from parthenolide-treated glioblastoma cells was assessed by ELISA. ELISA analysis showed that parthenolide decreased the protein levels of VEGF and MMP-9 in the culture media of glioblastoma cell. Representative data are shown for both assays. * indicates p < 0.0001.

We found that parthenolide treatment of glioblastoma cells significantly attenuates VEGF and MMP-9 mRNA expression (Figure 7A) and reduces the secretion of VEGF and MMP-9 protein into the culture medium (Figure 7B; *, p < 0.0001).

### Parthenolide suppresses neovascularisation and xenograft tumour growth

**Figure 8:**
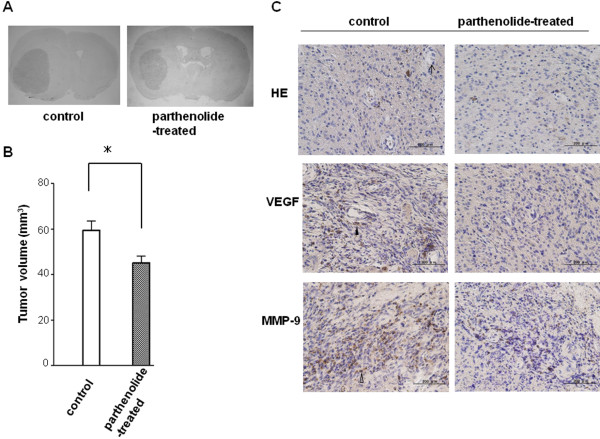
**Xenograft mouse model.** The effect of parthenolide on in vivo glioblastoma growth was determined using an intracerebral glioblastoma xenograft mouse model. (**A**) Representative coronal section of mouse brain implanted by glioblastoma cells. (**B**) Tumour volume analysis revealed that mean tumour volume of the parthenolide group (45.2 ± 2.74 mm^3^) was significantly smaller than that of the control group (59.5 ± 3.80 mm^3^). * indicates p < 0.0001. (**C**) In immunohistochemical stainings VEGF and MMP-9 can be depicted by brown DAB staining (VEGF-positive cell; *arrow head*, MMP-9-positive cell; *white arrow head*. Histological evaluation showed that the number of microvessel (*arrow*) and the expression of VEGF and MMP-9 was significantly lower in the tumours from parthenolide-treated and control mice (p < 0.0001). Bar = 200 μm.

Xenograft tumour volume was calculated from sequential histological sections (Figure 8A), and revealed that the mean tumour volume in parthenolide-treated mice (45.2 ± 2.74 mm^3^) is significantly smaller than in control animals (Figure 8B; 59.5 ± 3.80 mm^3^; *, p < 0.0001). Histological evaluation of HE staining showed that the mean MVD in parthenolide-treated mice (4.34 ± 0.89) is significantly lower than in control mice (Figure 8C; 6.52 ± 0.95; p < 0.0001). Moreover, VEGF and MMP-9 expression is reduced in the xenografts of parthenolide-treated mice relative to the controls (Figure 8C).

## Discussion

NF-κB plays a pivotal role in tumourigenesis and tumour progression [5,6]. Aberrant or constitutively activated NF-κB has been detected in human glioblastomas [15,16]. The sesquiterpene lactone parthenolide inhibits NF-κB by preventing the degradation of IκB-α and IκB-β [17]. We investigated the anti-invasive and anti-angiogenic effects of parthenolide on glioblastoma cells for the first time by using two PTEN-mutant glioblastoma cell lines.

Glioblastoma invasion into normal brain tissues involves disruption of the extracellular matrix (ECM) and the subsequent penetration of tumour cells into adjacent brain structures. This process is partly mediated by the tumour secretion of MMPs, a group of enzymes necessary for ECM degradation and involved in reconstructing ECM components and accelerating tumour cell migration [18]. Through their function as proteolytic enzymes, MMPs destroy the collagen components of the basement membrane (BM) and the ECM, thus disrupting intracellular adhesion, enabling tumour cell invasion, and promoting tumour progression. Elevated levels of MMPs, especially MMP-2 and MMP-9, correlate with glioblastoma tumour aggressiveness, and are believed to play an important role in tumour cell invasion [18-21]. In vivo and in vitro studies have revealed that the inhibiting MMP expression significantly reduces the invasive capacity of glioblastoma cells [22]. Dimerisation of the NF-κB transcription factor at the κB sequence in the MMP-9 promoter initiates MMP-9 transcription, thus upregulating MMP-9 protein expression [23]. Therefore, blocking NF-κB transcriptional activity by parthenolide may inhibit glioblastoma cell invasion. We found that parthenolide treatment inhibits both glioblastoma cell invasion and MMP-9 expression. These data suggest that parthenolide inhibition of glioblastoma cell invasion is mediated by NF-κB inhibition.

Tumour-induced angiogenesis is critical for the growth of solid tumours. Numerous studies have reported a correlation between increased intratumour microvessel density (MVD) and the risk of metastasis and/or decreased survival of patients with solid tumours [24-26]. NF-κB is an important upstream regulator of VEGF [27], a major angiogenic factor that induces endothelial cell proliferation. Glioblastomas are characterised by a high level of angiogenesis, and both VEGF secretion from tumour cells and TNF-alpha-induced NF-κB activation in endothelial cells promote endothelial cell survival by increasing anti-apoptotic gene expression under conditions of serum starvation. These pathways may, therefore, contribute to the maintenance of glioblastoma angiogenesis [28]. Aberrant MMP-9 expression is also implicated in the glioblastoma angiogenesis process. Low basal MMP-9 expression occurs in normal brain tissue, but high expression levels are induced in glioblastomas, and are linked to increased tumour cell proliferation [29]. We found that parthenolide suppresses both tumour cell-induced angiogenesis and expression of the NF-κB targets, i.e., VEGF and MMP-9, in glioblastoma cells. These data suggest that parthenolide suppresses tumour-induced angiogenesis through NF-κB inhibition.

With regard to tumor proliferation, we observed that parthenolide inhibits glioblastoma cell proliferation in dose-dependent manner. In the present study, cell viability (MTT assay/48h) of U87MG cells decreased to 85 % and 45 %, after the addition of 5 and 10 μM parthenolide, respectively, compared to the control. Zanotto-Filho et al. also reported that parthenolide inhibits glioblastoma cell proliferation in dose-dependent fashion and that NF-κB inhibition by parthenolide significantly correlated in decreases in cell viability of glioblastoma cells [12]. In their report, cell viability (MTT assay/36h) of U138MG cells decreased to 88 % and 34%, after the addition of 5 and 25 μM parthenolide, respectively, compared to the control. Our results were close to their research data. On the contrary, Anderson et al. performed proliferation assay using not the MTT but the CCK8 reagent. They reported that cell viability (CCK8 assay/24h) of U87MG cells decreased to about 78% and about 68%, after the addition of 5 and 10 μM parthenolide, respectively, compared to the control and that the inhibitory effect reached the plateau even if the concentration of parthenolide was raised exceeding 20 μM [11]. And they also reported that parthenolide was only able to suppress NF-κB activity by 20-30%. The reason why the inhibitory effect reached the plateau was not clarified. On the other hand, we agree with their opinion that another pathway other than NF-κB is also involved in antitumor effect of parthenolide.

By the way, NF-κB mediates the expression of anti-apoptotic and pro-apoptotic proteins. And most studies have attributed parthenolide suppression of tumour cell proliferation to the induction of apoptotic proteins mediated by NF-κB inhibition. In colorectal cancer cells, for example, conformational changes in Bax and upregulation of Bak lead to mitochondrial dysfunction and the induction of apoptosis [30]. Recent reports suggest that multiple pathways are involved in parthenolide-induced apoptosis in human cancer cells, including oxidative stress, intracellular thiol depletion, endoplasmic reticulum stress, caspase activation, and mitochondrial dysfunction [31-33]. In addition, parthenolide inhibition of c-Jun N-terminal kinase (JNK) sensitises JB6 murine epidermal cells to ultraviolet B (UVB)-induced apoptosis, suggesting an anti-apoptotic role for JNK [34]. Another report suggests that parthenolide- induced transcriptional suppression of pro-apoptotic genes is mediated by STAT inhibition and acts at both the transcriptional level and by direct inhibition of IKK-β [35].

In the present study, we used U87MG and U373 glioblastoma cells containing constitutively activated NF-κB due to PTEN mutation to examine cell survival and apoptosis. We demonstrated that parthenolide inhibits Akt phosphorylation, upregulates Bak and Bax, and activates caspase-3 and caspase-9. These results suggest that parthenolide induces apoptosis in glioblastoma cells by activating the mitochondrial apoptosis cascade and reducing survival signals through the inhibition of the Akt pathway. Parthenolide was previously reported to induce apoptosis in colorectal cancer and cholangiocarcinoma cells via the mitochondrial pathway [30,36]. In contrast, NF-κB inhibition by parthenolide markedly enhances the sensitivity of resistant breast cancer tumour cells to tamoxifen through suppression of the Akt pathway [37]. Taken together, the pro-apoptotic effect of parthenolide includes both stimulating the intrinsic apoptotic pathway and modulating expression of Bcl-2 family proteins.

In our in vivo study, parthenolide inhibited the growth of transplanted glioblastoma cells in mouse xenografts. This suggests that inhibitory effect of parthenolide on PTEN-mutant glioblastoma cells is caused by a combination of three mechanisms: suppression of tumour cell invasion, suppression of angiogenesis, and induction of tumour cell apoptosis.

We investigated tumour-induced angiogenesis by using a new in vitro angiogenesis assay that measures EC tube formation in collagen gels. Tumour-induced angiogenesis is usually examined by adding tumour cell medium directly to ECs without direct contact between the endothelial and tumour cells. In contrast, the new method allows the exchange of secreted factors between the endothelial and tumour cells. HUVECs have been used for many angiogenesis studies, especially those including in vitro experiments. However, microvascular endothelial cells from the organ being studied are presumed to be the most appropriate tool for tumour angiogenesis assays. Therefore, for the first time, we investigated the effect of parthenolide on angiogenesis by using HBMECs.

The effect of parthenolide treatment on neuro-inflammatory disorders has been examined in the recent years [38-40]. Runmel et al. investigated the potential of parthenolide to reduce brain inflammation and reported that parthenolide can cross the blood–brain barrier [40]. Thus, the combined antitumour and anti-inflammatory properties of parthenolide make it a promising candidate for further studies of neurological diseases.

## Conclusion

We report that parthenolide inhibits tumour progression in PTEN-mutant glioblastoma cells in vitro and in vivo, and suggest that parthenolide has a therapeutic potential as an antitumour agent for glioblastoma.

## Competing interests

The authors declare that they have no competing interests.

## Authors’ contributions

HN was responsible for the study design, interpretation of the data and revision of the manuscript. KS supervised the studies and helped to revise the manuscript. Both authors read and approved the final manuscript.

## Pre-publication history

The pre-publication history for this paper can be accessed here:

http://www.biomedcentral.com/1471-2407/12/453/prepub
